# Targeting Survivin Enhances Chemosensitivity in Retinoblastoma Cells and Orthotopic Tumors

**DOI:** 10.1371/journal.pone.0153011

**Published:** 2016-04-06

**Authors:** Angela Ferrario, Marian Luna, Natalie Rucker, Sam Wong, Ariel Lederman, Jonathan Kim, Charles Gomer

**Affiliations:** 1 The Saban Research Institute, Children’s Hospital Los Angeles, Los Angeles, California, 90027, United States of America; 2 Department of Ophthalmology, Keck School of Medicine, University of Southern California, Los Angeles, California, 90027, United States of America; 3 Departments of Pediatrics and Radiation Oncology, Keck School of Medicine, University of Southern California, Los Angeles, California, 90027, United States of America; Swedish Neuroscience Institute, UNITED STATES

## Abstract

Treatments for retinoblastoma (Rb) vary depending on the size and location of the intraocular lesions and include chemotherapy and radiation therapy. We examined whether agents used to treat Rb induce a pro-survival phenotype associated with increased expression of survivin, a member of the inhibitor of apoptosis family of proteins. We document that exposure to carboplatin, topotecan or radiation resulted in elevated expression of survivin in two human Rb cell lines but not in normal retinal pigmented epithelial (RPE) cells. Cellular levels of survivin were attenuated in Rb cells exposed to an imidazolium-based survivin suppressant, Sepantronium bromide (YM155). Protein expression patterns of survivin in RPE cells were not altered following treatment protocols involving exposure to YM155. Including YM155 with chemotherapy or radiation increased levels of apoptosis in Rb cells but not in RPE cells. Intraocular luciferase expressing Rb tumors were generated from the Rb cell lines and used to evaluate the effects of carboplatin and YM155 on in-vivo survivin expression and tumor growth. Carboplatin induced expression of survivin while carboplatin combined with YM155 reduced survivin expression in tumor bearing eyes. The combination protocol was also most effective in reducing the rate of tumor regrowth. These results indicate that targeted inhibition of the anti-apoptotic protein survivin provides a therapeutic advantage for Rb cells and tumors treated with chemotherapy.

## Introduction

Retinoblastoma (Rb) is the most common pediatric intraocular malignancy with an incidence of approximately 1 in 17,000 births [1]. The heritable form of Rb accounts for 40% of all cases, usually occurs during the first year of life, and most often presents as bilateral disease. The sporadic form of the disease accounts for the remaining 60% of cases, normally occurs between ages 2 and 5, and usually presents in only one eye [1–3]. Treatments for Rb depend on the size and location of the lesions and include local ablative procedures for small lesions and chemotherapeutic protocols for advanced disease [2]. External beam radiotherapy and plaque brachytherapy are now primarily used following disease recurrence. There is also an increased risk of secondary tumors following radiation in patients with the hereditary form of Rb [3]. Chemotherapy regimens include the intravenous administration of carboplatin, vincristine and etoposide and are combined with focal laser consolidation (i.e. chemoreduction) [3]. Additional chemotherapeutic agents, including topotecan, and single agent carboplatin plus novel drug delivery methods such as subconjunctival, ophthalmic artery, and intravitreal administration, are emerging as clinical options [4–7]. The overall survival of Rb patients and the tumor responsiveness of current treatments are excellent. However, tumor recurrences frequently lead to enucleations and there are ongoing concerns regarding both short and long term side effects from current therapies [6–11].

Survivin is a member of the inhibitor of apoptosis proteins (IAP) [12]. This 16.5 kDa protein is expressed in numerous cancers but is absent or minimally present in most terminally differentiated normal tissues [12]. Survivin is involved in a variety of cellular functions including cell cycle progression and inhibition of apoptosis [13]. Increased levels of survivin are associated with poor prognosis, cell proliferation, and metastasis in many malignancies [14,15]. Elevated tumor survivin levels are also associated with chemotherapy and radiation resistance [16,17]. Exposure of pancreatic and gastric tumor cells to chemotherapy and radiation therapy can increase survivin expression [18,19]. Survivin is reported in the aqueous humor and serum of Rb patients [20].

A variety of approaches, including antisense oligonucleotides, siRNA, ribozymes, immunotherapy, and small molecular weight inhibitors, are being tested in survivin targeting protocols with the goal of improving tumor treatment outcomes [21]. Knockdown of survivin expression by siRNAs enhance chemosensitivity and induces apoptosis in prostate and breast cancer cell lines [22,23]. The imidazolium-based agent, Sepantronium bromide (YM155), exerts anti-tumor activity via inhibition of survivin expression at both the mRNA and protein levels [24,25]. YM155 sensitizes non-small cell lung cancer cells to radiation and platinum-based drugs and enhances docetaxel toxicity of human melanoma cells via down-regulation of survivin expression and increased apoptosis [26–28]. In addition, YM155 also promotes DNA damage and autophagy [29]. Clinical trials using YM155 with and without chemotherapeutic agents have been reported [30–32]. The survivin suppressant has been well tolerated and studies have not identified normal ocular tissue toxicity.

In the current study we hypothesize that Rb treatments induce a pro-survival phenotype associated with increased survivin expression and targeting survivin with YM155 enhances the efficacy of current Rb treatments. Experiments examined the role of YM155 in modulating the expression of survivin as well as apoptosis in Rb and retinal pigmented epithelial (RPE) cells following treatment with carboplatin, topotecan or ionizing radiation. We also used orthotopic intraocular luciferase expressing Rb tumors to evaluate the effects of carboplatin and YM155 on in-vivo survivin expression and tumor growth. Our results indicate that targeted inhibition of the anti-apoptotic protein survivin provides a therapeutic advantage for Rb cells and tumors.

## Materials and Methods

### Drugs

Sepantronium bromide, YM155, (1-(2-methoxyethyl)-2-methyl-4,9-dioxo-3-(pyrazin-2-ylmethyl)-4,9-dihydro-1H-naphtho[2,3-d] imidazolium bromide), was purchased from Selleck Chemical Co, LTD (Houston, TX). The drug was dissolved in ddH_2_O to make a 10 μM stock solution and frozen at -20°C until used. Stock solutions were diluted to a working concentration of 2 nM in culture medium prior to cell culture experiments. For in-vivo experiments, YM155 was dissolved in ddH_2_O to make a 2.5 mg/ml stock solution. The drug was diluted 1 to 10 in saline and administered IP to achieve a 2 mg/kg treatment dose. Carboplatin was purchased from Teva Parenteral Medicines, Inc. (Irvine, CA). A stock solution at 10 mg/ml was stored at room temperature, and diluted to a working concentration of 5 μM in culture medium immediately prior to cell culture experiments. For in-vivo experiments, an aliquot of the stock solution was administered IP to mice to achieve a 60 mg/kg treatment dose. Topotecan hydrochloride was purchased from APP Pharmaceuticals, LLC (Schaumburg, IL). The drug was dissolved in ddH2O to make a 10 μM stock solution and frozen at -20°C until used. Stock solutions of topotecan were diluted to a working concentration of 10 nM in culture medium prior to cell culture experiments. D-luciferin was purchased from Promega Scientific (Madison, WI), dissolved in sterile PBS to make a 5 mg/ml stock solution, and kept at -20°C until used.

### Cell Lines and Treatment Protocols

Human Rb (Y79) cells (ATCC number HTB-18) and human retinal pigmented epithelial (RPE) cells (ATCC number CRL-2240) were obtained from American Type Culture Collection (Manassas, VA). A primary human Rb cell line (CHLA-215), obtained at the time of enucleation, was provided by the Children’s Oncology Group Cell Culture/Xenograft Repository. Western immunoblot analysis for Rb, phospho-Rb, and survivin expression confirmed that Rb and phospho-Rb expression was observed only in the RPE cells while basal survivin expression was observed in all cell lines (S1 Fig). Y79 cells were cultured in RPMI 1640 medium supplemented with 10% fetal bovine serum (FBS) and antibiotics (100 U/ml penicillin and streptomycin). CHLA-215 cells were cultured in IMDM medium supplemented with 20% FCS, ITS (insulin, transferrin, and selenium) culture supplement, and antibiotics. RPE cells were cultured in DMEM/F-12 medium supplemented with 20% FBS and antibiotics. Firefly luciferase expressing Y79 and CHLA-215 Rb cells (Y79-Luc and CHLA-215-Luc) under the control of a CMV promoter were obtained by transducing parental cells with the pCCL-c-MNDU3c-LUC packaged lentiviral vector (gift from Robert Seeger, Children’s Hospital Los Angeles) [33]. Cells were cloned by serial dilution in 96 well plates and checked for luciferase expression by bioluminescence imaging following administration of 25 μg D-luciferin. Clones with high levels of bioluminescence were selected, grown out, and used to produce Y79-Luv and CHLA-215-Luc xenografts.

### In Vitro Treatments and Assays

Cells were seeded in either 96 well plates to measure cytotoxicity or 100 mm Petri dishes to monitor apoptosis and protein expression. Twenty-four hours after seeding, cells were exposed to ionizing radiation (5 Gy) from a Cs-137 gamma irradiator, carboplatin (5 μM) or topotecan (10 nM). Cells were exposed to carboplatin or topotecan for 48 hours. When indicated, the cells were also incubated with YM155 (2 nM) for 72 hours, starting at the time they were seeded into culture plates or dishes. YM155, carboplatin and topotecan remained in the culture media until the cells were collected and analyzed for survival, apoptosis or protein expression. Cell viability was evaluated using a WST-1 (2-(4-iodophenyl)-3-(4-disulfophenyl)-2H-tetrazolium, monosodium salt) cell proliferation assay (Roche Applied Science, Indianapolis, IN) as per manufacturer’s instructions. Plates were read using a MR600 microplate reader (Dynatech, Golden Valley, MN) at 450 nM. The percentage of apoptotic cells was determined by FACS analysis (S2 Fig) using the FITC Annexin V Dead Cell Apoptosis Assay Kit (Thermo Scientific, Waltham, MA) as per manufacturers instructions. Caspase-3 activity was measured using the Caspase-3 Colorimetric Assay Kit, catalog number BF3100, from R&D Systems, Inc., Minneapolis, MN. Protein expression profiles were documented by Western immunoblot analysis [34]. Cells were collected at specified times and sonicated in 1X cell lysis buffer (Cell Signaling Technology, Danvers, MA) containing 1μM phenylmethylsulfonyl fluoride (Sigma, St. Louis, MO). Samples were size-separated on discontinuous gradient polyacrylamide gels (8–16%) and transferred overnight to nitrocellulose membranes. Filters were blocked for 1 h with 5% nonfat dry milk and then incubated overnight with either mouse monoclonal anti-survivin (Santa Cruz Biotechnology, Santa Cruz, CA), or rabbit polyclonal antibody to x-linked inhibitor of apoptosis (XIAP). Filters were then incubated with either an anti-mouse or an anti-rabbit peroxidase conjugate (Sigma) and the resulting complexes were visualized by enhanced chemiluminescence autoradiography (Amersham Life Science, Chicago, IL). Protein loading was evaluated by incubating the same filters with a mouse monoclonal anti-actin antibody (MP Biochemicals, Aurora, OH). Autoradiographs were quantified by scanning densitometry.

### In-vivo Treatments

Y79-Luc (2 X 10^3^) and CHLA-215-Luc cell clones (4 x 10^4^) in 4 μl of RPMI medium were injected into the posterior ocular chamber of the right eye of Nu/Nu mice (Jackson Laboratory, Bar Harbor, ME) using sterile 25 μl glass syringes fitted with 30 gauge needles (Hamilton, Reno, NV). Prior to each procedure, mice underwent inhalation anesthesia using isoflurane, and all efforts were made to minimize suffering. Tumor size was monitored by bioluminescence imaging following the ip administration of D-luciferin (150 mg/kg) using a cooled IVIS Animal Imaging System (Xenogen, Alameda, CA) coupled to the Living Image 2.5 software. Imaging was initiated 15 minutes after D-luciferin injection. Luminescence was expressed in photons/sec/cm^2^/steradian (FLUX). Treatments were initiated 14 days post inoculation or when the ocular luminescence signal was a minimum of 4 X 10^4^ p/sec/cm^2^/sr for Y79-Luc and CHLA-215-Luc lesions. Treatments consisted of three weekly cycles of carboplatin alone (60 mg/kg via ip injection delivered on days 2 and 5 of each cycle), YM155 alone (2 mg/kg via ip injection delivered on days 1 through 5 of each cycle), or a combination of carboplatin and YM155 administered as described above. Control mice received saline. Mice were examined weekly using bioluminescence imaging to document tumor growth. Representative bioluminescent images are shown for Y79-Luc and CHLA-215-Luc treated tumors (S3 Fig). Tumor growth delay was documented for the fast growing Y79-Luc lesions by plotting tumor flux (size) versus days post treatment. Long-term tumor response was documented for the slower growing CHLA-215-Luc lesions by plotting the percent animals with tumor luminescence values less than 1.2 X 10^5^ photons/sec/cm^2^/steradian. This value represented growth following treatment but was below the allowed tumor volume requiring animal sacrifice. Tumors that did not show growth by 90 days post treatment were considered cured. A second set of mice were sacrificed after one treatment cycle and the tumor bearing eye enucleated, homogenized with a polytron in 1X cell lysis buffer, and analyzed by Western immunoblot for protein expression as described above. All animal experiments were performed according to a protocol approved by the Institutional Animal Care and Usage Committee of Children’s Hospital Los Angeles (CHLA-316-15).

### Statistics

ANOVA tests and Bonferroni posttests have been performed on our data. An ANOVA online calculator was provided by the College of St. Benedicts, St. John’s University: www.physics.csbsju.edu/stats/anova. The Bonferroni posttest online calculator for significance between specific groups was provided by Graph Pad: www.graphpad.com/quickcals/posttest1.cfm. Differences with *p* < 0.05 were considered significant.

## Results

### Survivin is increased in Rb cells following chemotherapy or radiation treatments and is suppressed by YM155

**Fig 1:**
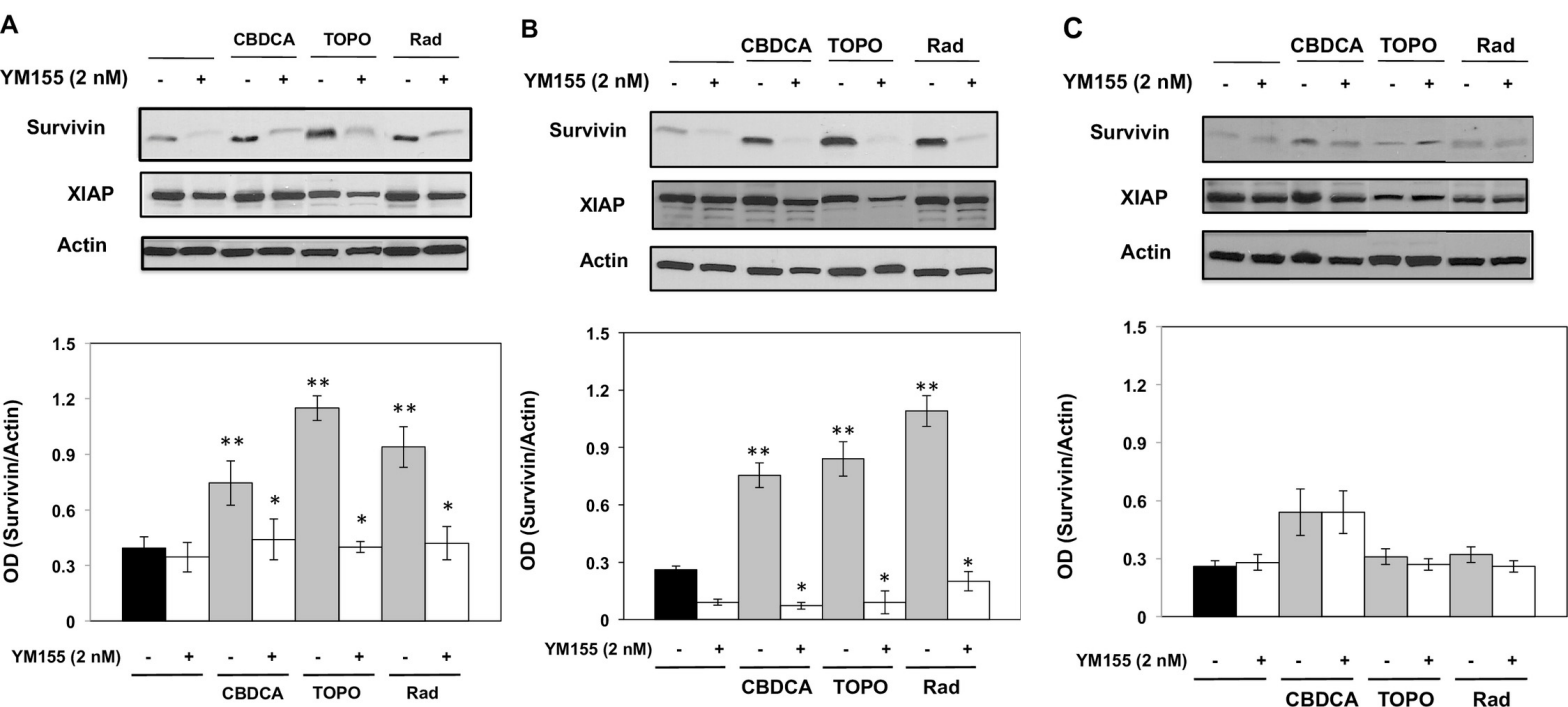
Treatment induced survivin expression. Survivin and XIAP expression profiles in Y79 (A), CHLA-215 (B), and RPE (C) cells exposed to carboplatin (CBDCA, 5 μM), topotecan (TOPO, 10 nM), or ionizing radiation (Rad, 5 Gy) with or without concomitant exposure to the survivin suppressant Sepantronium bromide (YM155, 2 nM). Cells were seeded on day 1 in growth medium in the presence or absence of 2 nM YM155 and 24 hours later cells were treated with or without CBDCA, TOPO, or Rad. At 72 hours, cells were harvested for protein expression analysis. Top figures show a representative Western immunoblot and bottom figures provides quantitative densitometric values from all immunoblots. *Columns*, mean; *bars*, ± SE of three to five separate experiments. ** *P* < 0.05 (Control vs CBDCA, TOPO, or Rad); *P < 0.05 (Control, CBDCA, TOPO or Rad in the absence of YM155 vs Control, CBDCA, TOPO or Rad in the presence of YM155).

We first examined survivin expression profiles in Rb and RPE cells exposed to chemotherapeutic agents or radiation with or without concomitant exposure to the survivin suppressant YM155. We also examined the expression profile of XIAP, another member of the family of IAPs. Fig 1A (upper panel) shows representative Western immunoblots of survivin and XIAP protein expression, and Fig 1A (lower panel) shows mean quantitative densimetric levels of survivin in Y79 Rb cells. Basal levels of survivin and XIAP were detectable in non-treated cells while exposure to carboplatin (5 μM), topotecan (10 nM), or radiation (5 Gy) only induced increased survivin expression. Co-incubation with YM155 decreased survivin levels in Y79 cells exposed to the chemotherapeutic treatments or radiation. Identical treatment parameters, using the primary Rb cell line CHLA-215, resulted in comparable expression profiles (Fig 1B). We next examined the effects of carboplatin, topotecan and radiation on ocular RPE cells. Fig 1C shows minimal effects of the chemotherapeutic agents or radiation, in the presence or absence of YM155, on survivin expression profiles for RPE cells. Expression of XIAP did not change after single or combined treatment protocols in either Rb or RPE cells and this agrees with reports indicating that YM155 preferentially targets survivin and not other members of the IAP family [26,27,35]. We did not observe changes in the subcellular distribution of survivin following treatments (S4 Fig). Baseline expression of survivin appears high in this figure and S1 Fig when compared to S5 Fig because of the extended film exposure time used for both S1 and S4 Figs. These results demonstrate that therapies to treat retinoblastoma induce a prosurvival phenotype associated with overexpression of survivin in Rb cells, and YM155 effectively attenuates expression of survivin in Rb cells while having minimal effect in ocular RPE cells.

### YM155 increases apoptosis and decreases viability in Rb cells following chemotherapy or radiation

**Fig 2:**
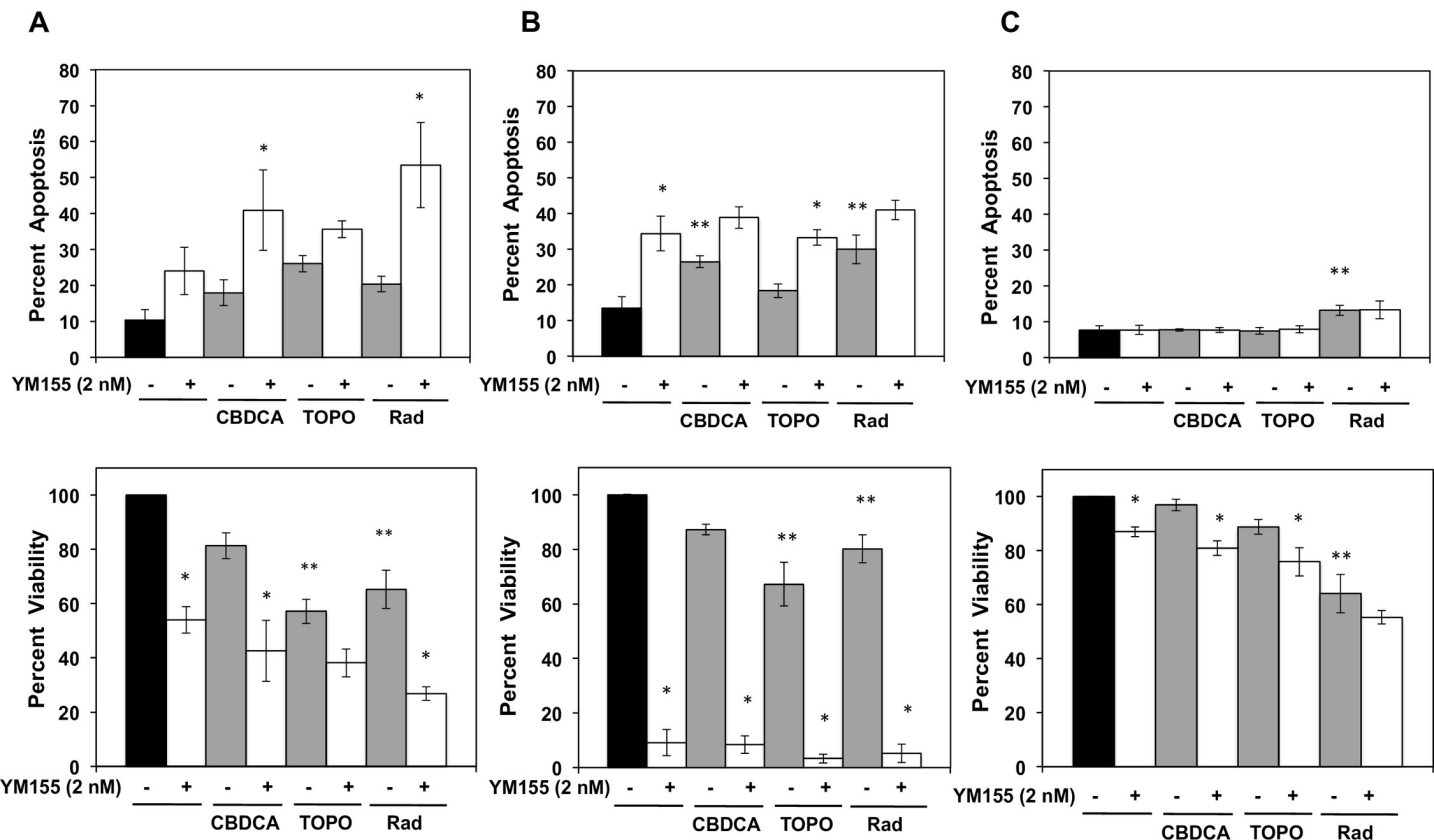
**YM155 enhances apoptosis and decreases viability in Y79 (A) and CHLA-215 (B) Rb cells exposed to chemotherapy or radiation.** YM155 decreased viability in RPE (C) cells and induced only a minimal apoptotic response in these cells following radiation. Percent apoptotic cells was determined by FACS analysis of annexin V and PI stained cells either under control or treatment conditions described in Fig 1. *Columns*, mean; *bars*, ± SE of three to five separate experiments. **P < 0.05 (Control vs CBDCA, TOPO, or Rad); *P < 0.05 (Control, CBDCA, TOPO or Rad in the absence of YM155 vs Control, CBDCA, TOPO, or Rad in the presence of YM155).

Fig 2 show apoptotic (upper panels) and viability levels (lower panels) in the 3 ocular cell lines (Y79, CHLA-215, and RPE) following exposure to YM155 alone and following exposure to carboplatin, topotecan, or radiation. The combination of YM155 with the individual chemotherapeutic agents or radiation further increased apoptotic levels in Y79 cells (Fig 2A). Apoptotic levels in CHLA-215 cells following combined treatments remained comparable to that induced by YM155 alone (Fig 2B). RPE cells (Fig 2C) exhibited a minimal basal level of apoptosis but did not undergo increased apoptosis following individual exposure to YM155 or chemotherapy (carboplatin or topotecan). A small increase in apoptosis was detected following radiation. We observed enhanced PARP cleavage in both Rb cell lines but not in RPE cells (S6 Fig). Caspase 3 activation was observed only in the Y79 cells (S6 Fig). Fig 2 (lower panel) also shows that cell viability, measured with the WST-1 assay, was significantly reduced when YM155 was combined with either chemotherapy or radiation in the Rb cell lines. We determined combination indexes using the method of Chou and Talalay (CompuSyn, Inc., computer program on Quantitation of Synergism and Antagonism in Drug Combinations) and viability data (S7 Fig and S1 Table). Synergism was observed in Y79 cells when YM155 was combined with carboplatin, topotecan, or radiation. For CHLA-215 cells, synergism was observed when YM155 was combined with topotecan or radiation, while for RPE cells synergism was observed when YM155 was combined with radiation. Lower YM155 doses for the CHLA-215 cells could alter Chou and Talalay synergy measurements because of the enhanced YM155 sensitivity of these cells.

### Survivin expression increases in orthotopic Rb tumors following carboplatin treatment

**Fig 3:**
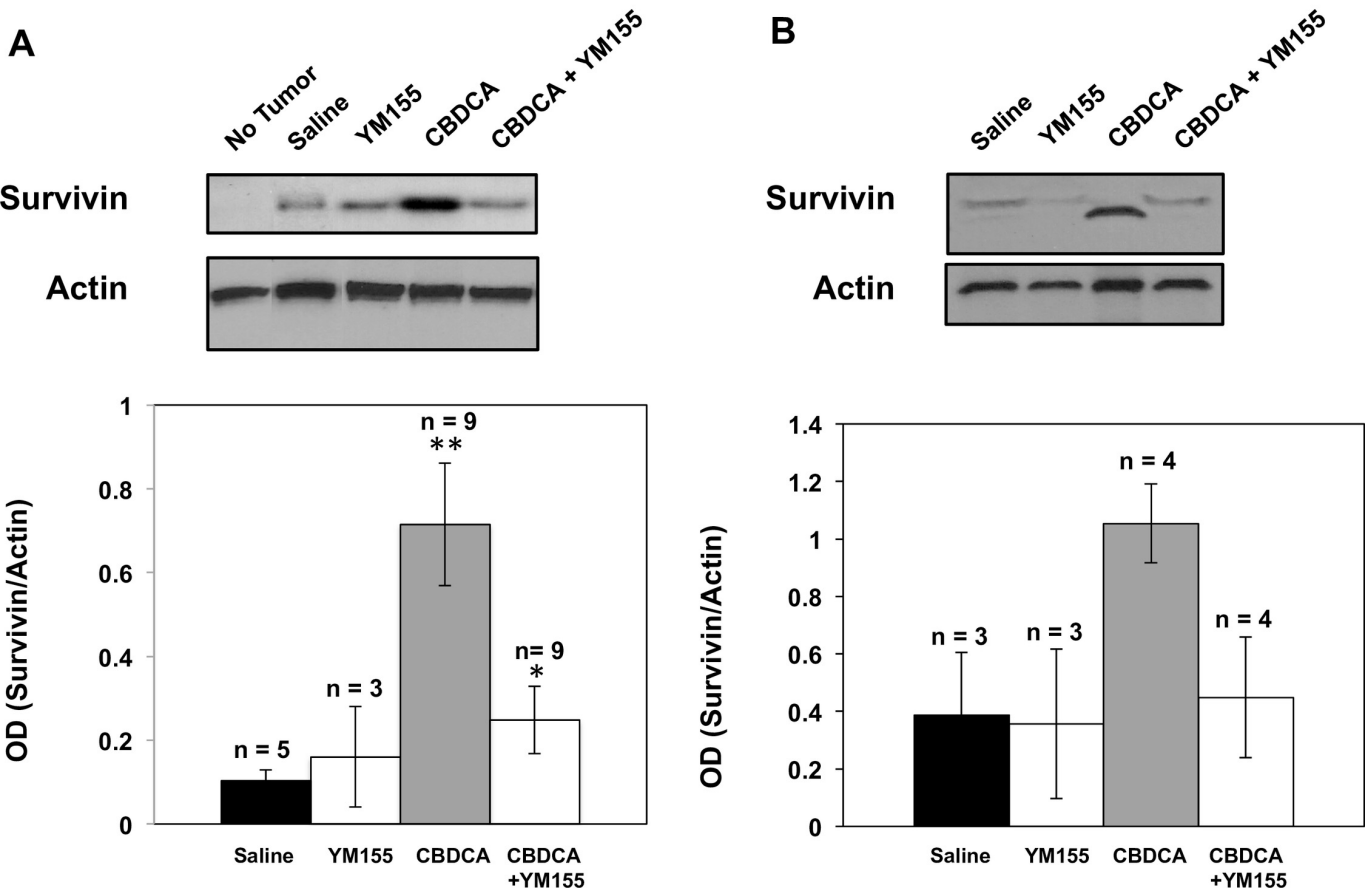
**Survivin expression profiles in Y79-Luc (A) and CHLA-215-Luc (B) tumors growing in the vitreal cavity of mice.** Mice were treated with carboplatin (CBDCA, 60 mg/kg) via IP injection on days 15 and 18 following transplantation, YM155 alone (2mg/kg) administered via IP injection for 5 consecutive days starting 14 days post transplantation, or the combination of CBDCA and YM155. Eyes were enucleated 24 hours following the last treatment, homogenized with a polytron in 1X cell lysis buffer, and analyzed for survivin expression by Western immunoblot and densitometry. Upper figure shows a representative Western immunoblot and lower figure provides quantitative densitometric values from all immunoblots. *Columns*, mean; *bars*, ± SE of three to nine separate intraocular lesions. **P < 0.05 (Control vs CBDCA). *P<0.05 (CBDCA alone vs CBDCA plus YM155).

We next examined if Rb tumors growing in the posterior chamber of mouse eyes also exhibited increased survivin levels when exposed to carboplatin, and whether systemic administration of YM155 reduced survivin expression. Y79 and CHLA-215 cells expressing the luciferase gene (Y79-Luc and CHLA-215-Luc) were transplanted to the posterior chamber of mouse eyes. Mice were treated with carboplatin alone (60mg/kg) via IP injection on day 15 and again on day 18 following transplantation, YM155 alone (2mg/kg) delivered via IP injection daily for 5 consecutive days starting on day 14, or the combination of carboplatin and YM155. Fig 3A shows representative survivin expression (upper panel) measured by immunoblot analysis and average densitometry measurements (lower panel) for mice with orthotopic Y79-Luc tumors. Fig 3B shows comparable results for mice with orthotopic CHLA-215-Luc tumors. Eyes were enucleated and processed 24 hours following the last treatment. Carboplatin treatment resulted in a significant increase in survivin expression within Rb tumors compared to mice with non-treated tumors. Eyes without tumors did not exhibit any survivin as expected. Mice receiving both carboplatin and YM155 had survivin levels that were reduced back to basal levels. Data for all examined mice is shown in S8 Fig. These results for orthotopic Rb tumors corroborate our in-vitro data showing increased survivin expression in Rb cells exposed to carboplatin as well as decreased survivin expression when carboplatin was combined with YM155. These observations demonstrate excellent cohesiveness between our in-vitro and in-vivo Rb experiments.

### YM155 enhances carboplatin responsiveness in orthotopic Rb tumors

**Fig 4:**
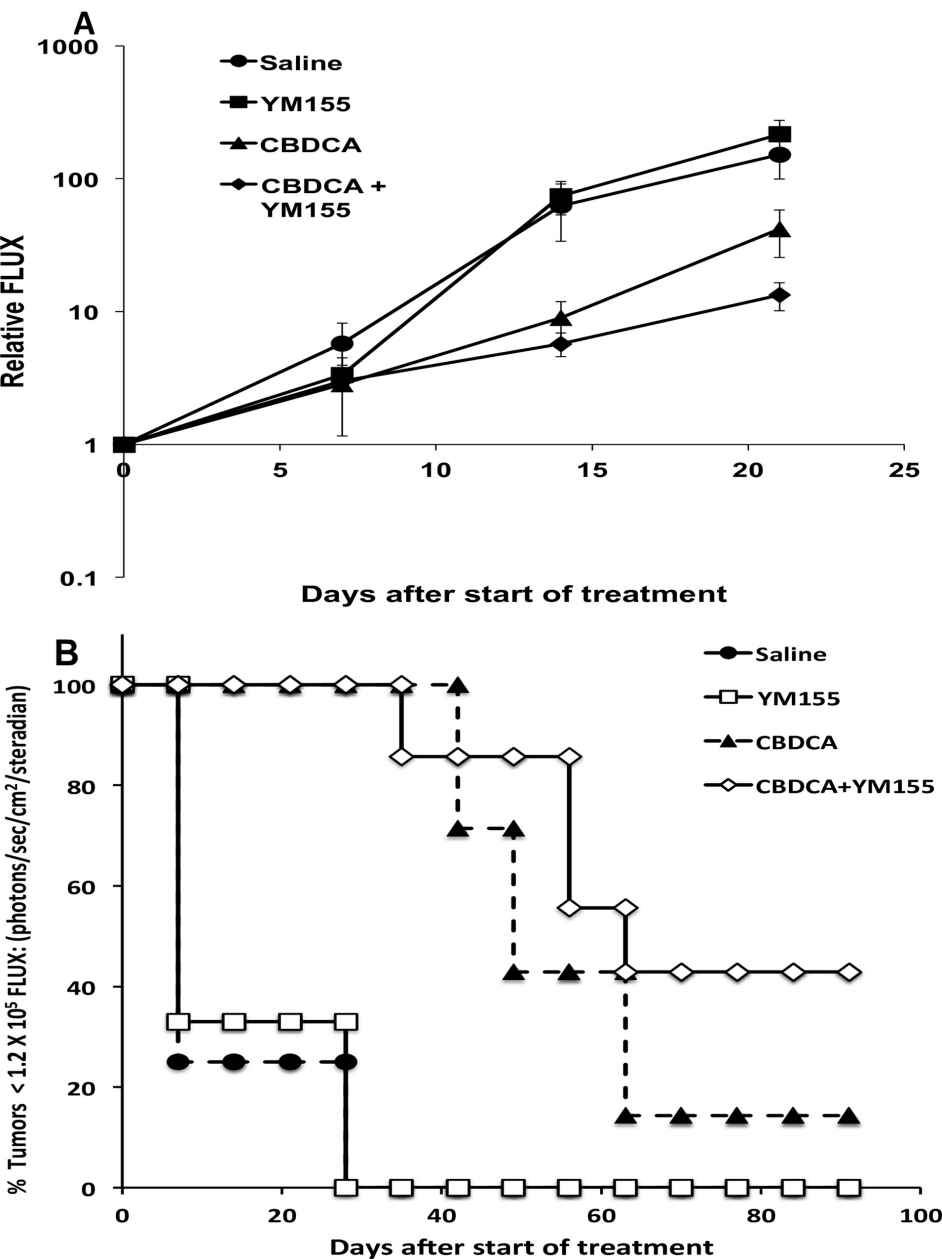
**Tumor responses for Y79-Luc (A) and CHLA-215-Luc (B) orthotopic tumors treated with YM155, CBDCA, or YM155 plus CBDCA.** Treatments consisted of three weekly cycles of CBDCA alone (60 mg/kg via ip injection delivered on days 2 and 5 of each cycle), YM155 alone (2 mg/kg via ip injection delivered on days 1 through 5 of each cycle), or a combination of CBDCA and YM155 administered as described above. Control mice received saline. All mice were examined weekly using bioluminescence imaging to document tumor growth. The bioluminescence values emitted from Y79-Luc ocular tumors are plotted as a function of time following treatment and this correlates with the rate of growth for the intraocular lesions. For the slower growing CHLA-215-Luc tumors, the percentage of tumors remaining at or below a basal level of bioluminescence flux (1.2 X 10^5^ photons/sec/cm^2^/steradian) was plotted as a function of time following treatment. N = 7–9 for treated lesions (CBDCA alone or CBDCA plus YM155), 3–8 for YM155 alone, and 4–6 for saline alone controls.

The tumoricidal effectiveness of combining YM155 with carboplatin was examined in our two orthotopic Rb tumor models. Fig 4A shows tumor grow kinetics measured by intraocular tumor induced bioluminescence flux for Y79-Luc lesions and Fig 4B shows length of time CHLA-215-Luc tumors remained below a basal tumor size or bioluminescence flux (1.2 X 10^5^ photons/sec/cm^2^/steradian). Growth of individual Rb tumors was measured weekly (S2–S4 Tables). The Y79-Luc tumor line grew at a faster rate than the CHLA-215-Luc tumor and yet both tumor lines exhibited comparable trends. Treatment of mice with YM155 alone resulted in comparable results to non-treated mice. Mice receiving carboplatin alone induced a measurable tumor growth delay or an extended time at a basal tumor size for the Y79-Luc and the CHLA-215-Luc tumors, respectively. The combination of carboplatin and YM155 resulted in a further delay in tumor growth compared to either carboplatin or YM155 alone. The in-vivo experiments with Y79 or CHLA-215 tumors show trends toward enhanced effects but are not statistically significant by ANOVA analysis followed by Bonferroni post test for tumors treated with carboplatin versus carboplatin plus YM155.

## Discussion

Primary treatment for Rb shifted from external beam radiotherapy to chemotherapy in large part to avoid the significant morbidity induced by ionizing radiation, including the induction of second cancers. Treatment responsiveness also increased with this paradigm shift but the chemotherapeutic agents currently used are associated with side effects such as neutropenia. Therefore less toxic protocols and new drug delivery methods continue to be evaluated [36,37]. Intraocular tumor recurrences also occur in a significant percentage of patients with current treatment approaches. Novel molecular targets are being examined for Rb with the objectives of decreasing side effects by dose reduction of chemotherapeutic agents as well as by preferentially enhancing Rb tumor cytotoxicity. Targeted molecules for Rb include the HDAC inhibitor suberoylanilide hydroxamic acid (SAHA) [38], and the AMP-kinase activator, 5-aminoimidazole-4-carboxamide-1-beta-4-ribofuranaside (AICAR) [39]. Additionally, MDMX and MDM2 proteins degrade the Rb and P53 protein products and a small molecule inhibitor of MDMX and MDM2, Nutlin 3A, is cytotoxic to Rb cells and exhibits further cytotoxicity when combined with topotecan [40].

Our study documents, for the first time, that targeting the pro-survival molecule, survivin, is an attractive option for Rb. We discovered survivin levels are increased in both established (Y79) and primary (CHLA-215) Rb cell lines exposed to carboplatin, topotecan, and radiation. We observed that a small molecule inhibitor of survivin, YM155, suppresses survivin in Rb cells following treatment, decreases Rb cell viability, and enhances apoptosis. We also demonstrated that survivin is induced when Y79 cells were exposed simultaneously to carboplatin and topotecan, and reduced when YM155 was included (S5 Fig). In addition, YM155 down regulated survivin induced by carboplatin in orthotopic Rb tumors, and the combination of carboplatin and YM155 enhanced in-vivo treatment responsiveness compared to single agent protocols.

The three treatments used in our in vitro experiments are relevant to clinical Rb protocols. Carboplatin is included in the chemoreduction protocol (carboplatin, etoposide and vincristine) for Rb with most patients receiving 6 monthly cycles of this regimen [11,41]. Carboplatin is also used as a single agent with novel ophthalmic delivery protocols for treating Rb [7]. Periocular topotecan is being investigated at a preclinical level for episcleral delivery and intravitreal administration of topotecan is being used to target Rb seeds [42–44], as well as in combination with carboplatin [45]. Radiation has a long history in Rb therapy but is now primarily used for intraocular relapse [46]. Our interest in survivin developed as we observed increased expression of this pro-survival molecule when Rb cells were exposed to these chemotherapeutic agents or radiation. Survivin is negatively regulated, in part, by the Rb protein and this may play a role in the up regulation of survivin and concomitant resistance observed in tumor cells with non-functioning Rb genes [47]. Little has been reported regarding survivin and Rb. Survivin is detected in the aqueous humor of Rb patients and levels correlate with tumor stage [20]. Rb cell proliferative activity is also positively correlated with survivin and HSP-90 expression in clinical Rb histological specimens [48]. Survivin expression in 42 archival Rb tumors has recently been reported with 86% (36/42) tumors showing survivin expression [49]. In contrast, RPE cells did not exhibit significant changes in survivin levels or apoptosis in our experiments and this agrees with a prior study reporting that RPE cells express low basal survivin and caspase-8 levels [50]. Viability assays also demonstrated that RPE cells were more resistant than Rb cells to carboplatin, topotecan, and YM155 (S9 Fig). In this case we used a 96 hr WST-1 time frame instead of the 48 hr WST-1 time frame used in Fig 2 and S7 Fig. Our results provide strong evidence for targeting survivin as a strategy for Rb treatment protocols.

A large number of laboratories continue to examine methods of targeting survivin as an adjunctive therapy in clinical and preclinical studies. Effective survivin inhibitors include antisense oligonucleotides, small molecule inhibitors, and survivin targeting via cell based immunotherapy based vaccines [51]. We chose to use the small molecule inhibitor, YM155, in our initial experiments. YM155 inhibits the survivin promoter and also induces direct DNA damage as measured by induction of gamma-H2AX and pKAP-1 expression in PC3 prostate cancer cells [52]. We also observed enhanced pKAP1 expression in YM155 treated Rb cells (data not shown). YM155 combined with paclitaxel and carboplatin was well tolerated but did not increase treatment responsiveness in a non-randomized phase I/II trial of advanced non-small cell lung cancer [53]. YM155 alone or in combination with an anti-CD52 monoclonal antibody, alemtuzumab, were effective in adult T-cell leukemia with the combination showing enhanced tumoricidal activity [54]. Clinical trials involving YM155 with docetaxel for stage III and IV melanoma, refractory prostate CA, and metastatic breast cancer are in progress [55]. YM155 has a short half-life, which may contribute to the minimal clinical responsiveness reported to date. A liposomal formulation of YM155 has been developed to address the short half-life. Xenografts of human prostate tumors show enhanced pharmacokinetics, with significantly increased peak YM155 levels and enhanced tumor and serum retention compared to the parent YM155 compound. However, the extended retention in kidney will need to be further evaluated for potential renal issues [56]. As regards pediatric tumors, a recent preclinical study shows that a panel of pediatric acute myeloid leukemia cells exhibit significant sensitivity to YM155 alone and in combination with cytarabine or daunorubicin via down regulation of survivin and concomitant increased apoptosis and DNA damage [57]. Promising new prosurvival targeting compounds are also being reported. Recently, a novel small molecule camptothecin derivative, FL118, was shown to strongly inhibit survivin as well as Mcl-1, XIAP, and CIAP2 and enhance tumoricidal responses in preclinical models [58].

In summary, we show YM155 increases radiosensitivity and chemo-sensitivity of Rb cells by inhibiting survivin and enhancing apoptosis while there was minimal effect on normal RPE cells. YM155 also enhanced the tumoricidal responsiveness of carboplatin in orthotopic Rb tumors growing in the posterior chamber of mouse eyes. Combination treatment employing a survivin inhibitor with currently used modalities may prove to be clinically beneficial by selectively increasing overall tumor kill or possibly lowering the dose of chemotherapeutic agents needed to effectively treat Rb lesions.

## Supporting Information

S1 Fig Western immunoblots showing Rb (BD Biosciences), phospho Rb (Cell Signaling) and survivin (Santa Cruz Biotechnology) expression in Y79, CHLA-215, and RPE cells. The Rb and phospho Rb proteins are observed only in RPE cells.
(TIF)

S2 Fig Representative flow cytometry of Y79, CHLA-215 and RPE cells. Cells were treated with either carboplatin (5 μM), topotecan (10 nM) or radiation (5 Gy) +/- YM155 (2 nM) and then assayed for apoptosis using the FITC Annexin V Dead Cell Apoptosis Assay kit stained with FITC Annexin V and propidium iodine (Thermo Scientific). Apoptotic values were calculated from the percentage of cells in the upper right (UR) and the lower right (LR) quadrants.
(TIF)

S3 Fig **Representative bioluminescent images for treated Y79-Luc tumors (A) and CHLA-215-Luc tumors (B).** When image fluxes reached 4 X 10^4^, mice received 3 weekly cycles of either carboplatin alone (60 mg/kg via ip on days 2 and 5 of each cycle), YM155 alone (2 mg/kg ip on days 1 through 5 of each cycle), or combination CBDCA and YM155 as described above. Control mice were administered saline on the same schedule as combination therapies.
(TIF)

S4 Fig **Survivin expression profiles in whole cells lysates as well as in cytoplasmic and nuclear fractions for Y79 (A), CHLA-215 (B) and RPE (C) cells exposed to carboplatin (CBDCA, 5** μ**M), topotecan (TOPO 10 nM), or ionizing radiation (Rad, 5 Gy) with or without concomitant exposure to YM155 (2 nM).** Cells were seeded on day 1 in growth medium in the presence or the absence of 2 nM YM155 and 24 hours later cells were treated with or without CBDCA, TOPO or Rad. At 4 hours (CBCDA, TOPO) or 1 hour (Rad), cells were harvested for whole cell, cytoplasmic and nuclear protein expression analysis (NE-PER Nuclear and Cytoplasmic Extraction Reagents, Thermo Scientific). Calnexin and Lamin B1 were used as subcellular markers for cytoplasm and nucleus, respectively.
(TIF)

S5 Fig Survivin, XIAP, and Actin protein expression in Y79 Rb cells following single agent (5 μM carboplatin or 10 nM topotecan) +/- 2 nM YM155, or double agent (carboplatin and topotecan) plus/minus YM155. The double agent exposure induced survivin expression and YM155 suppressed survivin expression.
(TIF)

S6 Fig Analysis of PARP cleavage and Caspase-3 activation in Rb and RPE cells. (A) Western immunoblot showing cleaved PARP (Asp214) via detection of a large 89kDa fragment of human PARP resulting from cleavage of aspartic acid 214. The antibody does not recognize full length PARP (cleaved PARP mouse mAb from Cell Signaling), and (B-D) caspase-3 activity determined using a commercial caspase-3 activity assay (R&D Systems) in Y79, CHLA-215, and RPE cells exposed to either 5 μM carboplatin, 10 nM topotecan, or 5 Gy radiation +/- 2 nM YM155. Panel A shows enhanced PARP cleavage by YM155 in the two Rb cell lines but not in the RPE cells. Panels B-D show enhanced caspase-3 activity by YM155 in Y79 cells but not in Rb CHLA-215 or RPE cells. Data represents one experiment in which each value is the average of 2 separate sample determinations.
(TIF)

S7 Fig Cell viability data for combination indexes to evaluate synergy. Twenty-four hours after plating, cells were exposed to ionizing radiation (5 or 10 Gy) from a Cs-137 gamma irradiator, carboplatin (5 or 10 μM) or topotecan (10 or 20 nM). Cells were exposed to carboplatin or topotecan for 48 hours. When indicated, the cells were also incubated with YM155 (2 nM) starting at the time they were seeded into culture plates or dishes. YM155, carboplatin and topotecan remained in the culture media until the cells were collected and analyzed for survival, 72 hr after plating. Mean +/- SE. N = 2–5 experiments with 8 replicates per dose per experiment.
(TIF)

S8 Fig Western immunoblot and densitometric values of survivin in Rb orthotopic tumors. Complete survivin expression analysis in Y79-Luc tumors (A) and CHLA-215-Luc tumors (B) growing in the vitreal cavity of mice. Individual Western immunoblots and average densitometric values are shown for each tumor. Mice were treated with carboplatin alone (CBDCA, 60 mg/kg) via IP administration on days 15 and 18 following tumor cell transplantation, YM155 alone (2 mg/kg) via IP injection for 5 consecutive days starting 14 days post transplantation, or a combination of CBDCA and YM155. Controls were administered saline for 5 days starting on day 14. Eyes were enucleated 24 hours after the last treatment, homogenized and analyzed via Western immunoblot analysis.
(TIF)

S9 Fig Survival curves for Y79, CHLA-215, and RPE cells exposed to carboplatin, topotecan, radiation or YM155. Viability was determined using the WST-1 assay. Treatment conditions: Twenty-four hours after seeding, cells were exposed to ionizing radiation from a Cs-137 gamma irradiator, carboplatin, topotecan, or YM155. YM155, carboplatin and topotecan remained in the culture media until the cells were collected and analyzed for survival, which was 96 hr after the start of treatment. Error bars are the mean +/- SE. N = 2 experiments with 8 replicates per dose point per experiment.
(TIF)

S1 Table Calculated combination indexes to evaluate synergy using the method of Chou and Talalay. Viability data used for the calculations in S7 Fig.
(TIF)

S2 Table Luminescence (photon/sec/cm^2^ steradian: flux) for mice bearing Y79-Luc tumors. Each line represents readings from an individual mouse.
(TIF)

S3 Table Relative flux calculations (luminescence: photon/sec/cm2 steradian or FLUX) for mice bearing Y79-Luc tumors. Individual fold increase in FLUX values for each mouse.
(TIF)

S4 Table Relative flux values (photons/sec/cm^2^/steradian) for CHLA-215-Lux tumors. Each line represents an individual mouse tumor. Values in bold represent tumors with bioluminescence flux at or below 1.2 X 10^5^ photons/sec/cm^2^/steradian.
(TIF)
