# Supplementary figures and images for: Targeting Survivin Enhances Chemosensitivity in Retinoblastoma Cells and Orthotopic Tumors
Source: PLoS One. 2016 Apr 6;11(4):e0153011. doi: 10.1371/journal.pone.0153011 (PMC4822873; doi:10.1371/journal.pone.0153011)

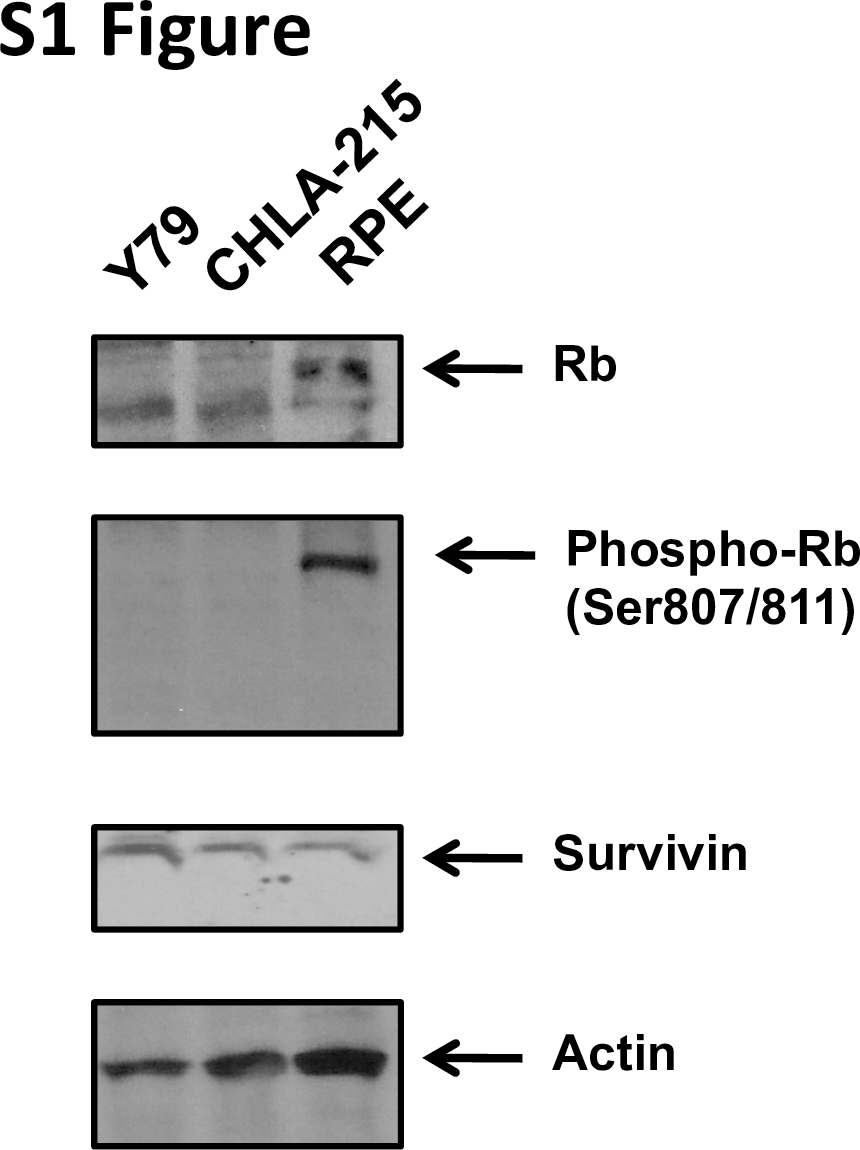

Supplement: S1 Fig — The Rb and phospho Rb proteins are observed only in RPE cells. (TIF) [file pone.0153011.s001.tif]

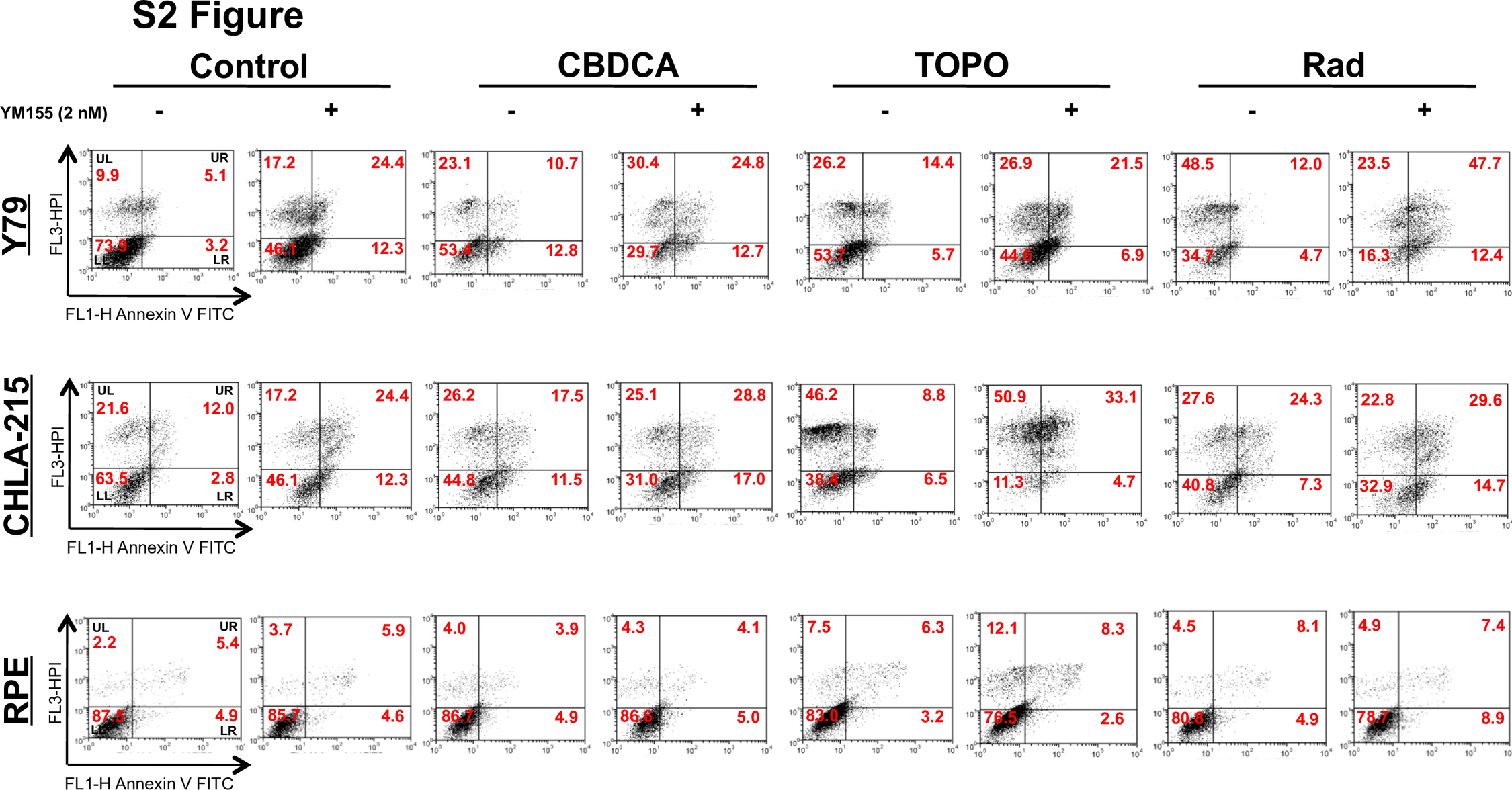

Supplement: S2 Fig — Cells were treated with either carboplatin (5 μM), topotecan (10 nM) or radiation (5 Gy) +/- YM155 (2 nM) and then assayed for apoptosis using the FITC Annexin V Dead Cell Apoptosis Assay kit stained with FITC Annexin V and propidium iodine (Thermo Scientific). Apoptotic values were calculated from the percentage of cells in the upper right (UR) and the lower right (LR) quadrants. (TIF) [file pone.0153011.s002.tif]

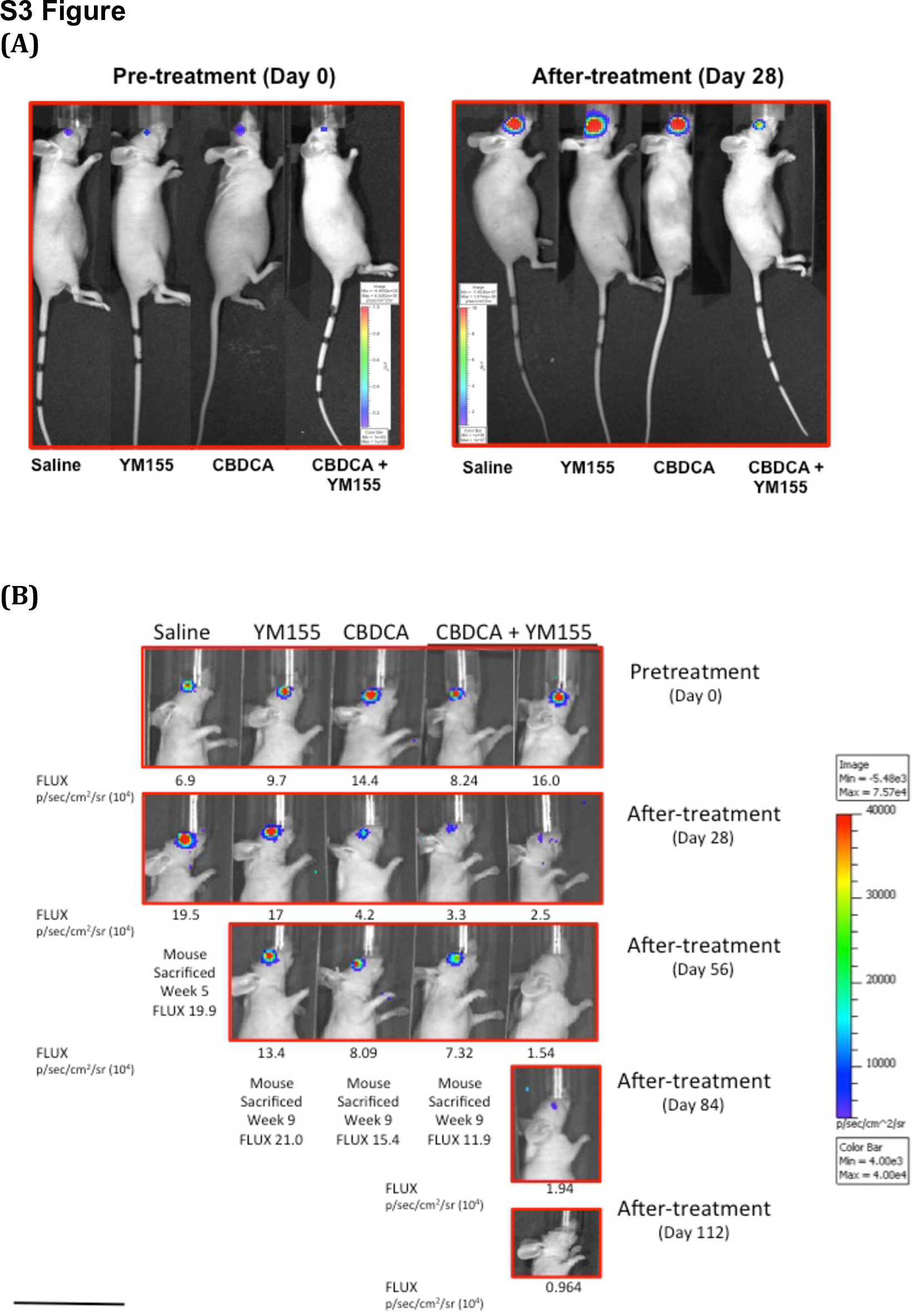

Supplement: S3 Fig — Representative bioluminescent images for treated Y79-Luc tumors (A) and CHLA-215-Luc tumors (B). When image fluxes reached 4 X 104, mice received 3 weekly cycles of either carboplatin alone (60 mg/kg via ip on days 2 and 5 of each cycle), YM155 alone (2 mg/kg ip on days 1 through 5 of each cycle), or combination CBDCA and YM155 as described above. Control mice were administered saline on the same schedule as combination therapies. (TIF) [file pone.0153011.s003.tif]

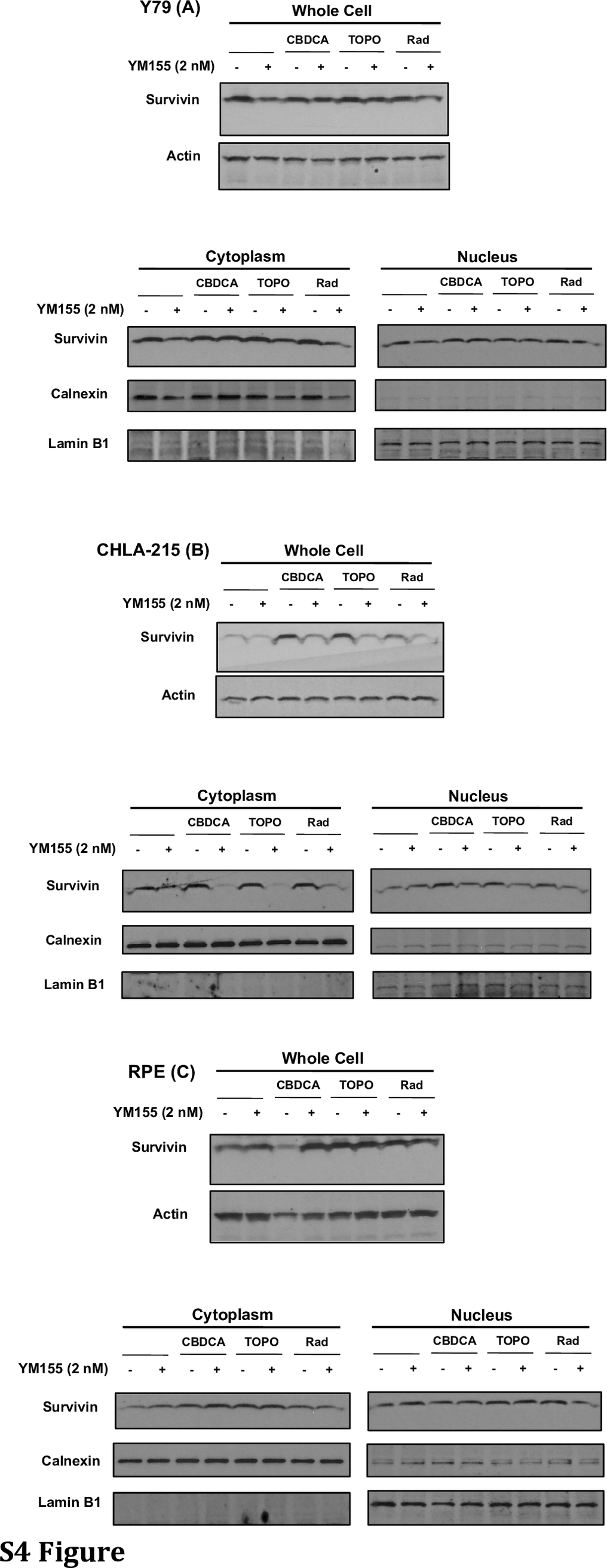

Supplement: S4 Fig — Survivin expression profiles in whole cells lysates as well as in cytoplasmic and nuclear fractions for Y79 (A), CHLA-215 (B) and RPE (C) cells exposed to carboplatin (CBDCA, 5 μM), topotecan (TOPO 10 nM), or ionizing radiation (Rad, 5 Gy) with or without concomitant exposure to YM155 (2 nM). Cells were seeded on day 1 in growth medium in the presence or the absence of 2 nM YM155 and 24 hours later cells were treated with or without CBDCA, TOPO or Rad. At 4 hours (CBCDA, TOPO) or 1 hour (Rad), cells were harvested for whole cell, cytoplasmic and nuclear protein expression analysis (NE-PER Nuclear and Cytoplasmic Extraction Reagents, Thermo Scientific). Calnexin and Lamin B1 were used as subcellular markers for cytoplasm and nucleus, respectively. (TIF) [file pone.0153011.s004.tif]

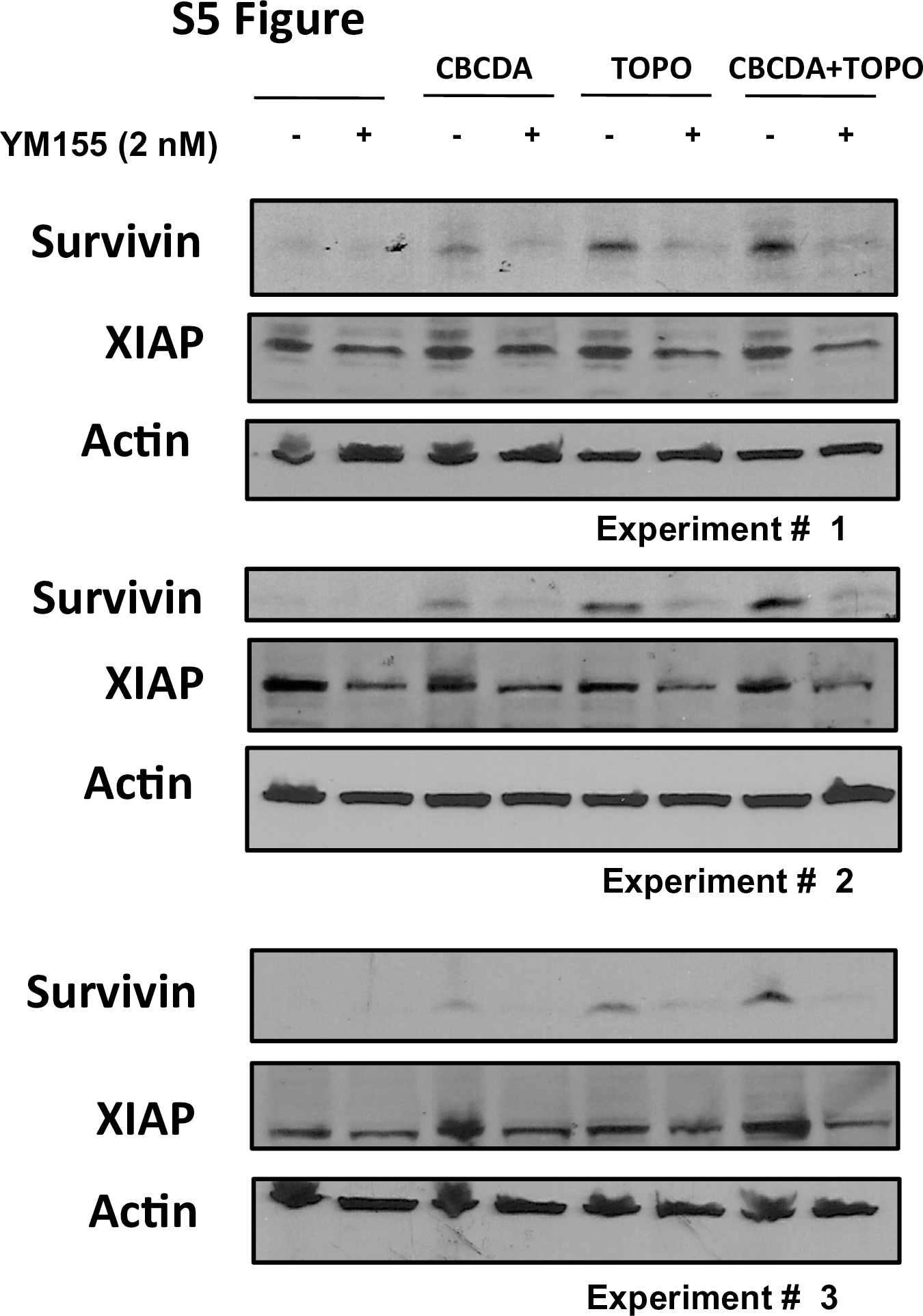

Supplement: S5 Fig — The double agent exposure induced survivin expression and YM155 suppressed survivin expression. (TIF) [file pone.0153011.s005.tif]

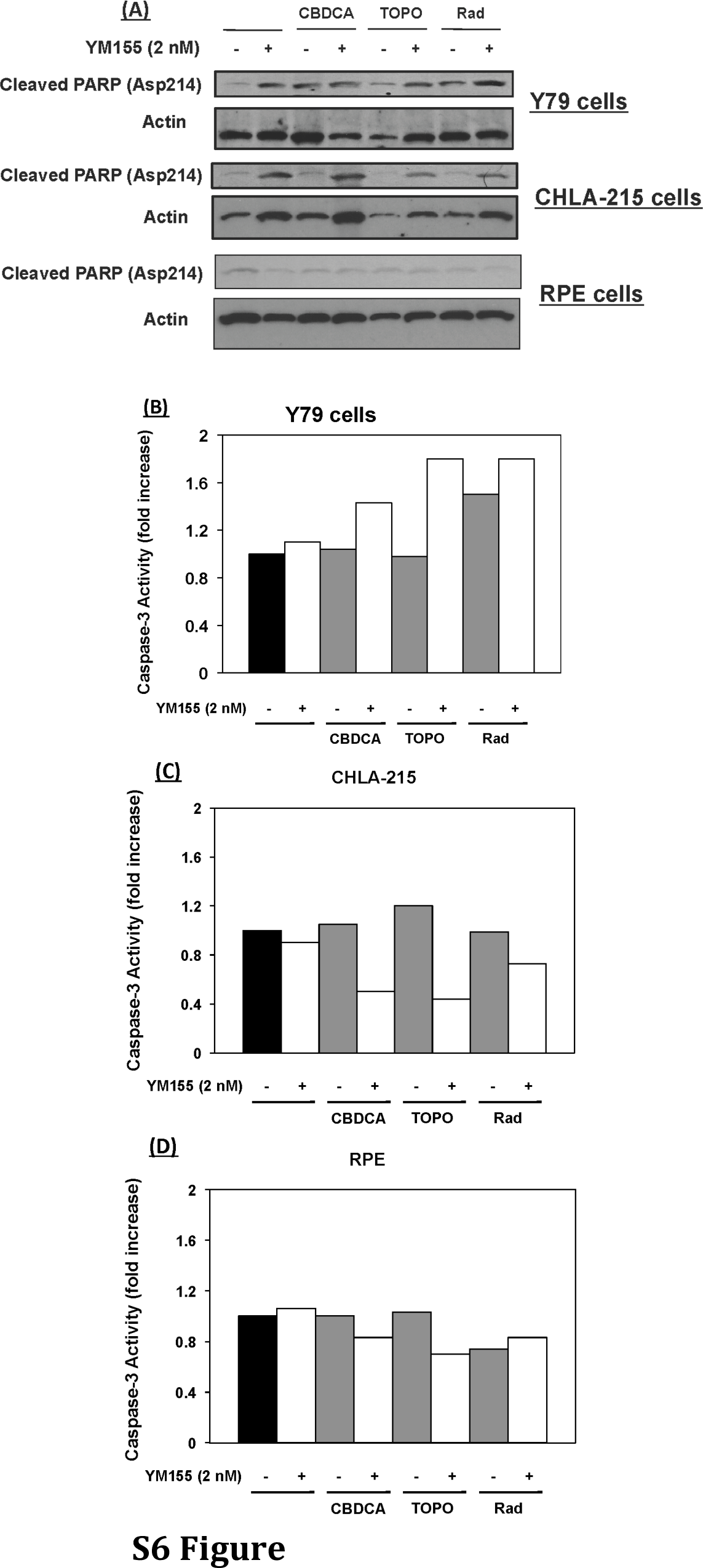

Supplement: S6 Fig — (A) Western immunoblot showing cleaved PARP (Asp214) via detection of a large 89kDa fragment of human PARP resulting from cleavage of aspartic acid 214. The antibody does not recognize full length PARP (cleaved PARP mouse mAb from Cell Signaling), and (B-D) caspase-3 activity determined using a commercial caspase-3 activity assay (R&D Systems) in Y79, CHLA-215, and RPE cells exposed to either 5 μM carboplatin, 10 nM topotecan, or 5 Gy radiation +/- 2 nM YM155. Panel A shows enhanced PARP cleavage by YM155 in the two Rb cell lines but not in the RPE cells. Panels B-D show enhanced caspase-3 activity by YM155 in Y79 cells but not in Rb CHLA-215 or RPE cells. Data represents one experiment in which each value is the average of 2 separate sample determinations. (TIF) [file pone.0153011.s006.tif]

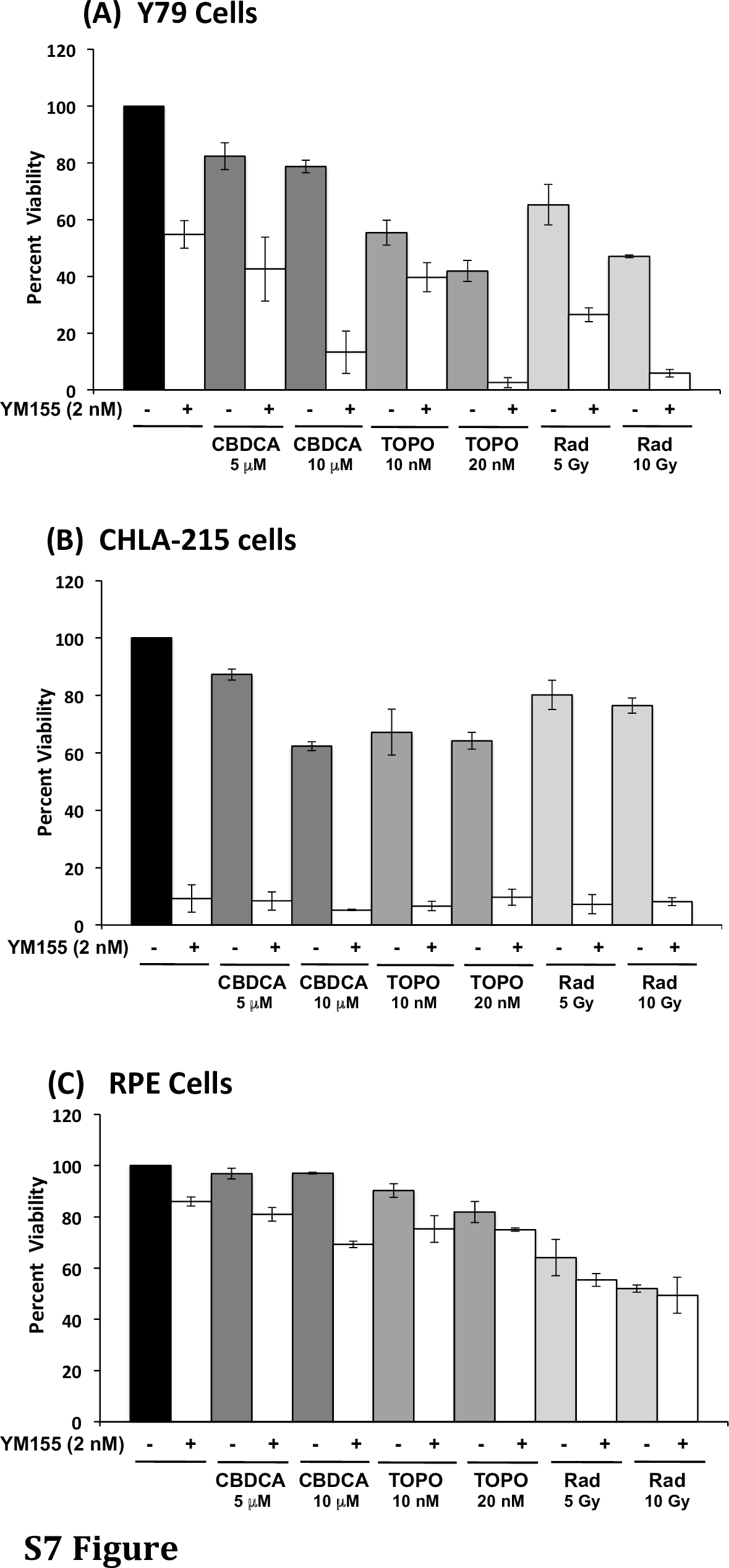

Supplement: S7 Fig — Twenty-four hours after plating, cells were exposed to ionizing radiation (5 or 10 Gy) from a Cs-137 gamma irradiator, carboplatin (5 or 10 μM) or topotecan (10 or 20 nM). Cells were exposed to carboplatin or topotecan for 48 hours. When indicated, the cells were also incubated with YM155 (2 nM) starting at the time they were seeded into culture plates or dishes. YM155, carboplatin and topotecan remained in the culture media until the cells were collected and analyzed for survival, 72 hr after plating. Mean +/- SE. N = 2–5 experiments with 8 replicates per dose per experiment. (TIF) [file pone.0153011.s007.tif]

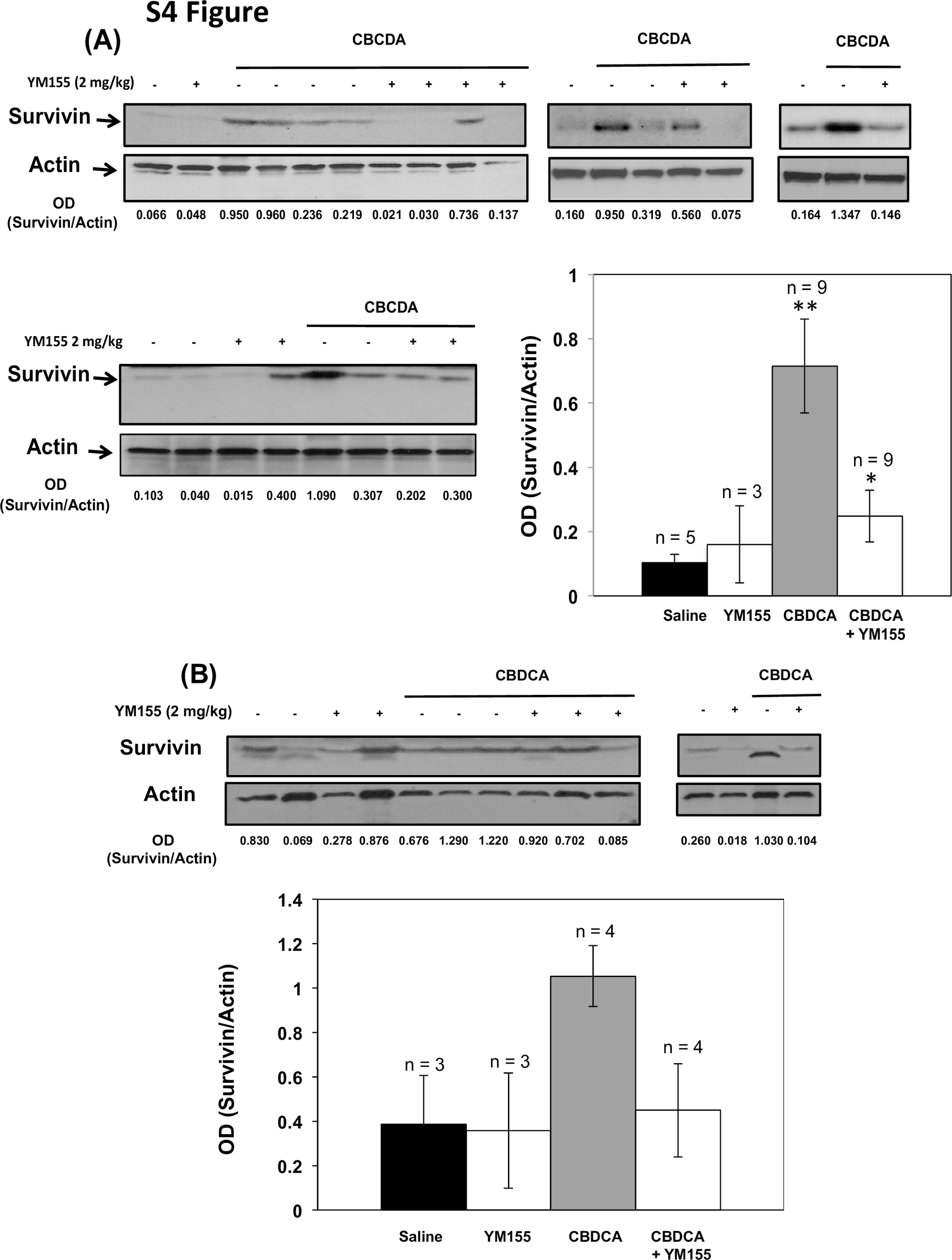

Supplement: S8 Fig — Complete survivin expression analysis in Y79-Luc tumors (A) and CHLA-215-Luc tumors (B) growing in the vitreal cavity of mice. Individual Western immunoblots and average densitometric values are shown for each tumor. Mice were treated with carboplatin alone (CBDCA, 60 mg/kg) via IP administration on days 15 and 18 following tumor cell transplantation, YM155 alone (2 mg/kg) via IP injection for 5 consecutive days starting 14 days post transplantation, or a combination of CBDCA and YM155. Controls were administered saline for 5 days starting on day 14. Eyes were enucleated 24 hours after the last treatment, homogenized and analyzed via Western immunoblot analysis. (TIF) [file pone.0153011.s008.tif]

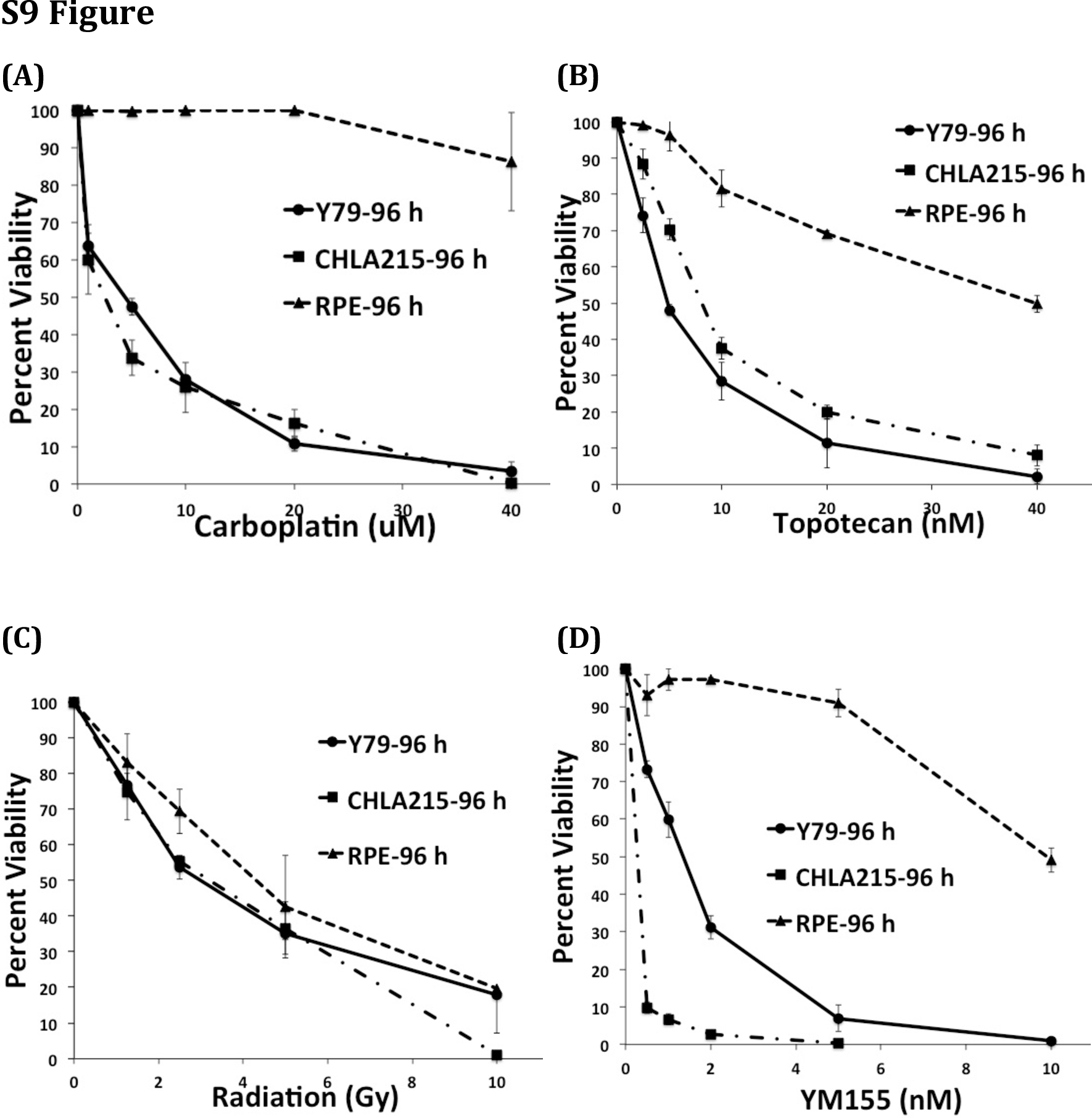

Supplement: S9 Fig — Viability was determined using the WST-1 assay. Treatment conditions: Twenty-four hours after seeding, cells were exposed to ionizing radiation from a Cs-137 gamma irradiator, carboplatin, topotecan, or YM155. YM155, carboplatin and topotecan remained in the culture media until the cells were collected and analyzed for survival, which was 96 hr after the start of treatment. Error bars are the mean +/- SE. N = 2 experiments with 8 replicates per dose point per experiment. (TIF) [file pone.0153011.s009.tif]

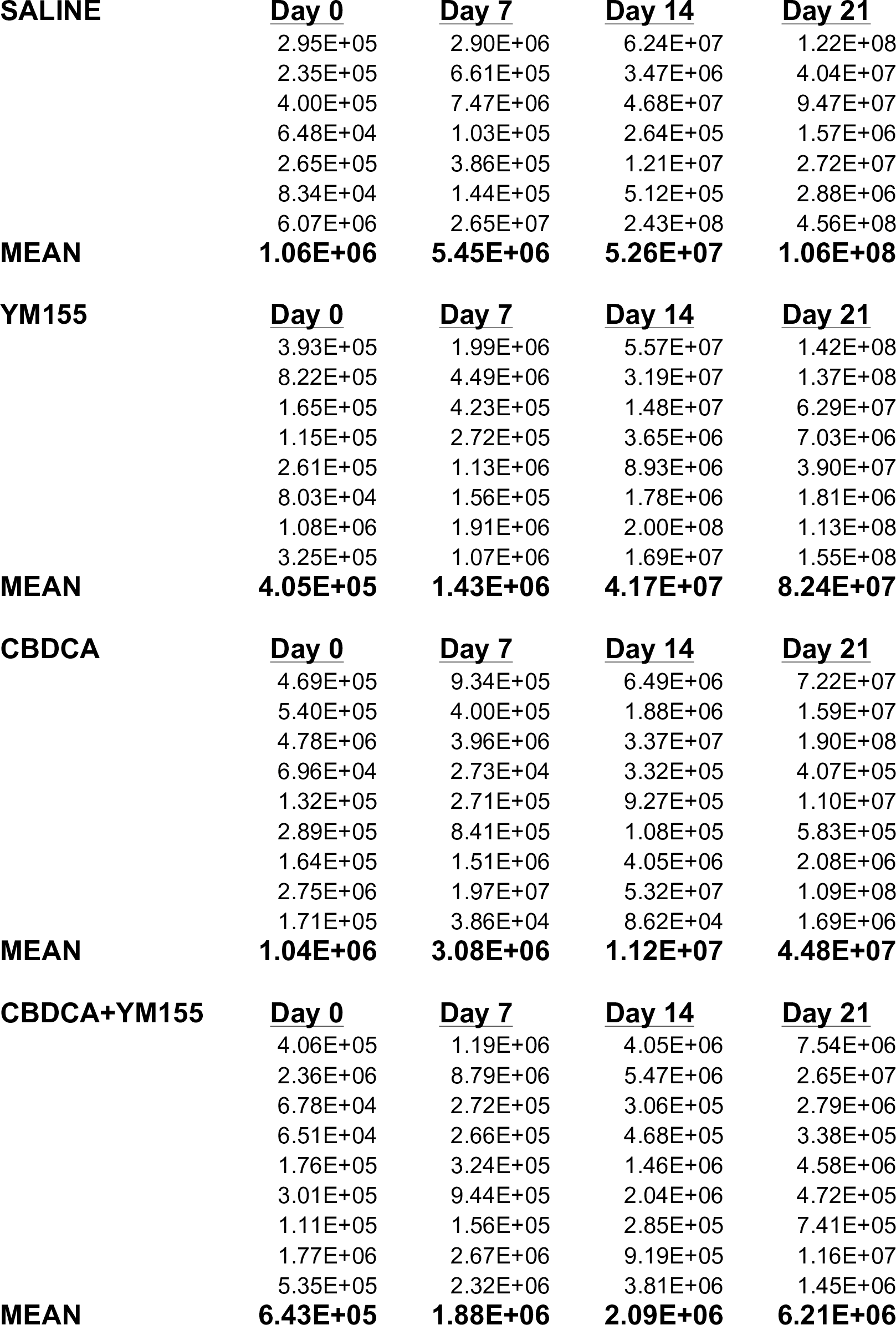

Supplement: S1 Table — Viability data used for the calculations in S7 Fig. (TIF) [file pone.0153011.s010.tif]

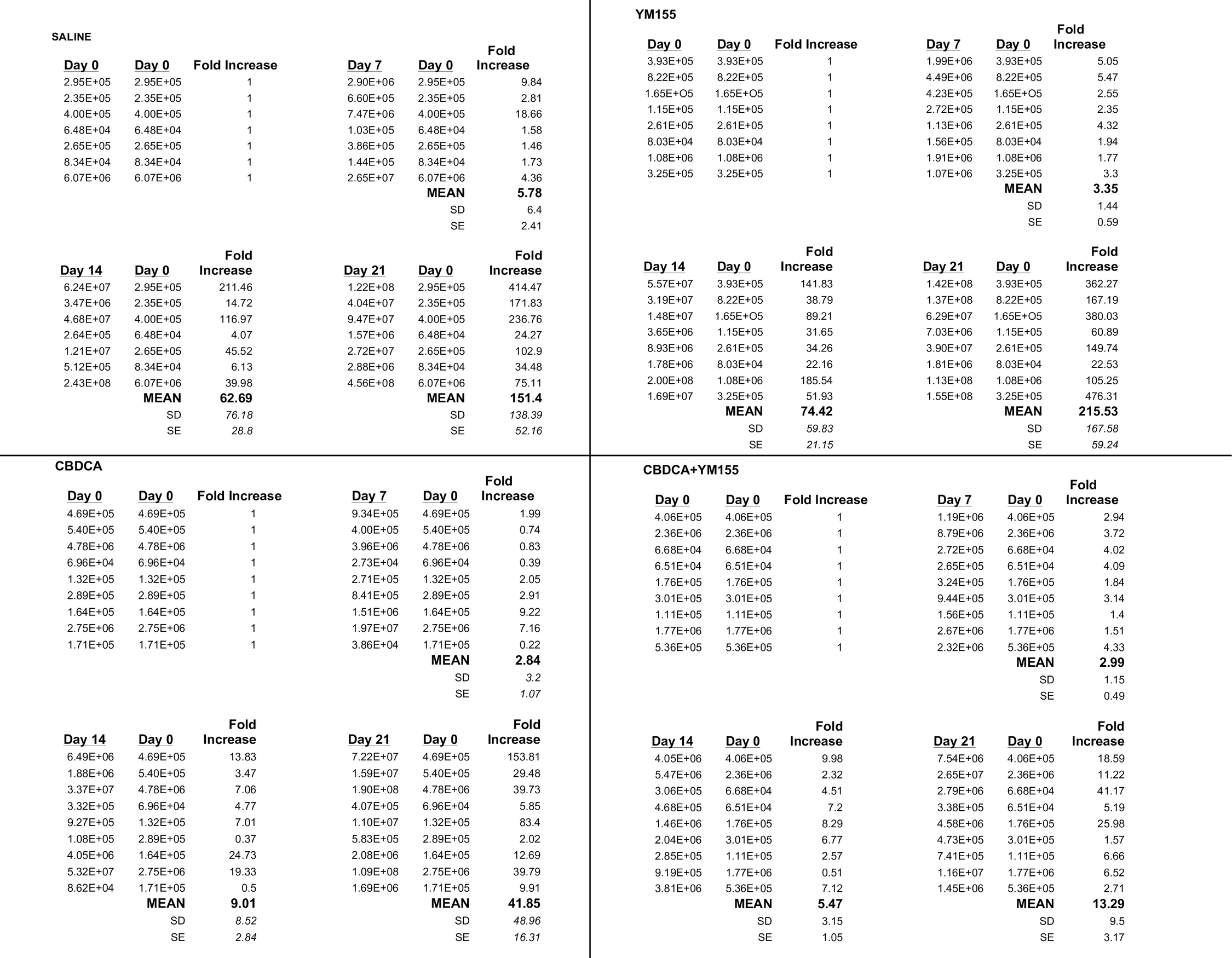

Supplement: S2 Table — Each line represents readings from an individual mouse. (TIF) [file pone.0153011.s011.tif]

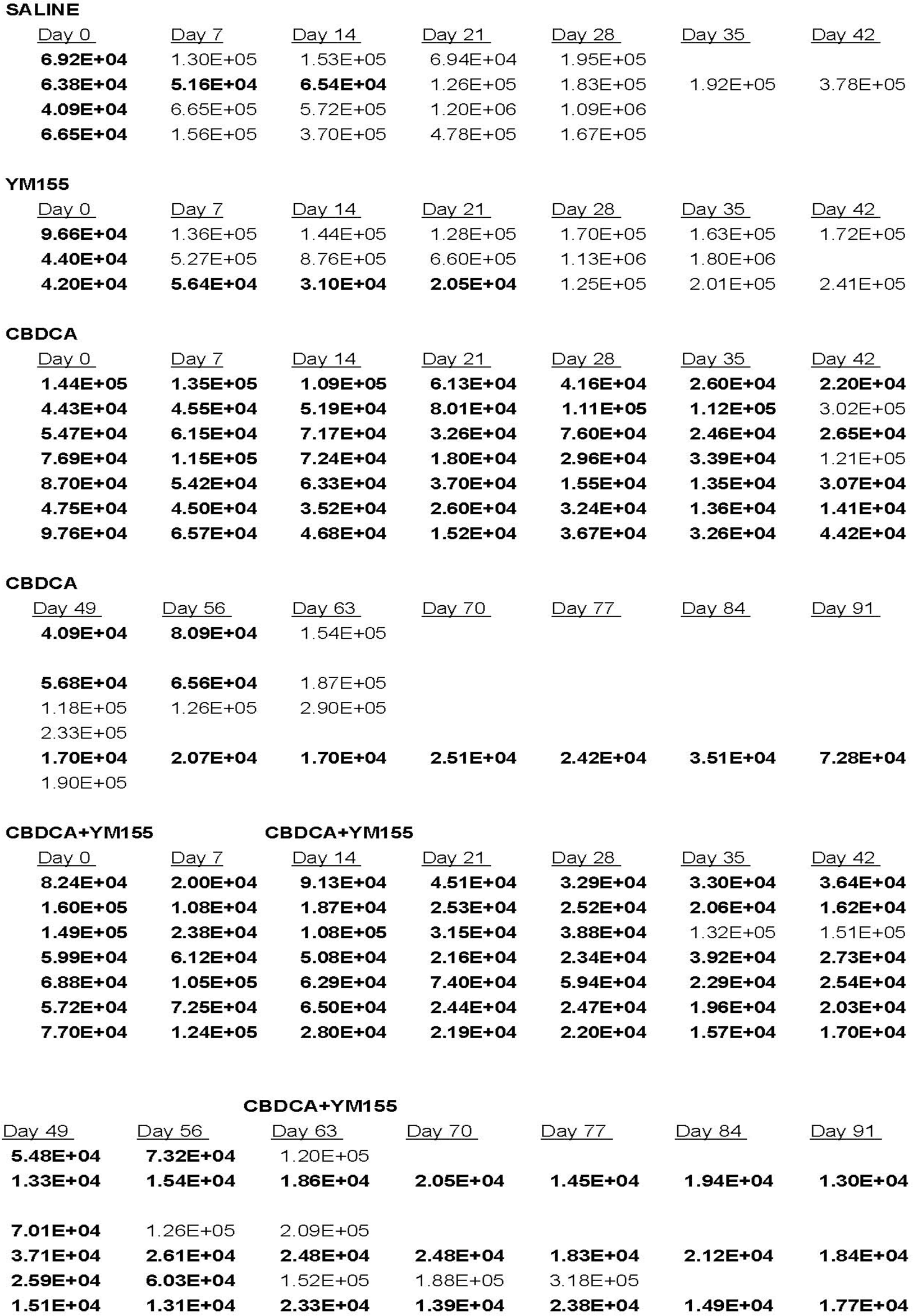

Supplement: S3 Table — Individual fold increase in FLUX values for each mouse. (TIF) [file pone.0153011.s012.tif]

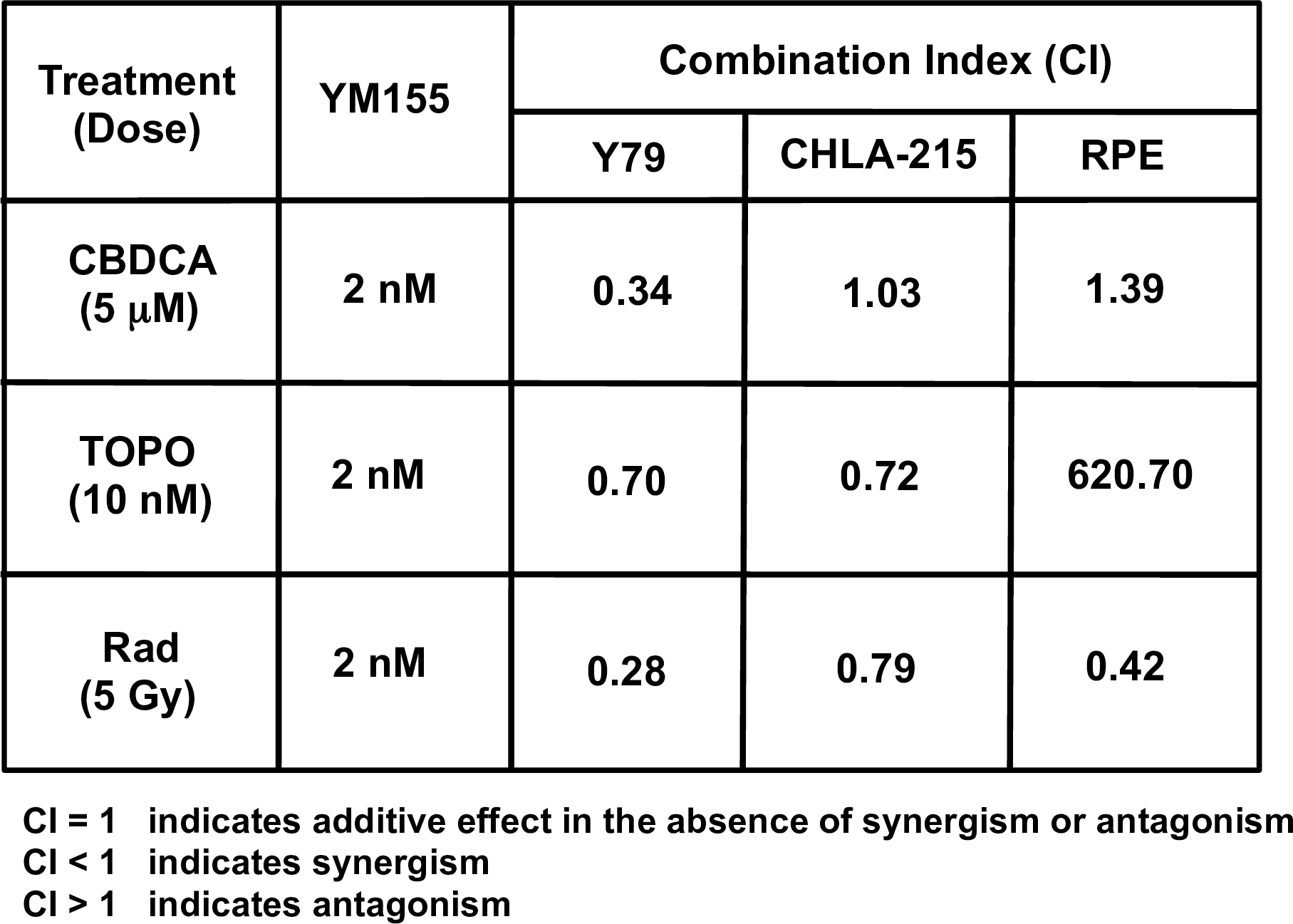

Supplement: S4 Table — Each line represents an individual mouse tumor. Values in bold represent tumors with bioluminescence flux at or below 1.2 X 105 photons/sec/cm2/steradian. (TIF) [file pone.0153011.s013.tif]
